# Modified Chaihu Shugan Powder for Functional Dyspepsia: Meta-Analysis for Randomized Controlled Trial

**DOI:** 10.1155/2013/791724

**Published:** 2013-05-13

**Authors:** Nan Yang, Xuehua Jiang, Xuelan Qiu, Zhiqiang Hu, Ling Wang, Minxian Song

**Affiliations:** ^1^Department of Clinical Pharmacy & Pharmacy Administration, West China Pharmacy School, Sichuan University, No. 17 Section 3 Renmin Nanlu, Chengdu, Sichuan 610041, China; ^2^School of Pharmacy, Jiangxi University of Traditional Chinese Medicine, No. 18, Yunwan Road, Wanli District, Nanchang, Jiangxi 330004, China

## Abstract

*Context*. Modified Chaihu Shugan powder (MCSP) is a popular traditional Chinese herbal formula for functional dyspepsia, which is revised from Chaihu Shugan San and recorded in a medical classic works of China. However, its role and effect in treating functional dyspepsia have not been well established. *Objective*. To assess the effect and safety of modified Chaihu Shugan powder for functional dyspepsia. *Methods*. We searched the published and unpublished studies up to August 2012. Only RCTs of modified Chaihu Shugan powder with or without prokinetic drugs versus prokinetic drugs in the patients diagnosed with functional dyspepsia were included. *Results*. Twenty-two clinical trials involving 1998 participants were included. There were evidences that modified Chaihu Shugan powder (RR = 1.20, 95%, CI 1.14 to 1.27) and modified Chaihu Shugan powder plus prokinetic drugs (RR = 1.18, 95%, CI 1.11 to 1.25) were significantly better treatment options than prokinetic drugs alone in improving symptoms. No serious adverse events were described in the included trials. *Conclusions*. This meta-analysis showed that modified Chaihu Shugan powder alone or in combination with prokinetic drugs might be more effective than prokinetic drugs alone. However, with poor methodological quality, all the included trials were at high risk of bias. Further large-scale high-quality trials are required for assessment.

## 1. Introduction

### 1.1. Rationale

Functional dyspepsia (FD), namely, functional gastrointestinal disorders or nonulcer dyspepsia, refers to symptoms centered in the upper abdominal region in absence of organic disease, such as epigastric pain, early satiety, fullness, belching, nausea, and vomiting [1–3]. It is a highly prevalent disorder. With influence of the definition applied, the global prevalence of FD had varied between 11.5% and 45% [4–6]. Although it is not a life-threatening condition, a number of out-patient studies suggested that FD markedly impaired patients' work and quality of life and laid a significant economic burden to the healthcare system [7–9]. Multiple factors, like motility abnormality, visceral hypersensitivity, psychosocial factors, excess secretion of gastric acid, duodenal acidity, helicobacter pylori, environment, diet, postinfectious factors, and genetics, were likely involved, but the pathogenesis of FD remains obscure [2, 6, 10, 11]. For this reason, no single medicine is effective for all patients with FD. In the area of medical therapy, traditional Chinese medicine (TCM) plays an important part, besides prokinetics, antacids, H_2_-receptor antagonists, proton pump inhibitors, helicobacter pylori eradication, and antidepressants [12]. It was reported that at least one-third the US population used some form of TCM on a routine basis [13].

Chaihu Shugan San (CSS) is a classical and effective prescription recorded in a medical classic, Jingyue Quanshu also known as Jingyue's Complete Works, written in Ming Dynasty (1368–1644 year) of China, which has been used to improve some symptoms similar to FD by soothing liver, regulating qi, and relieving pain according to TCM theory. CSS are composed of Chinese Thorowax, Rhizoma Cyperi, Szechwan Lovage Rhizome, Pericarpium Citri Reticulatae, Fructus Aurantii, white peony root, and licorice. As we know every formula of TCM is an organic whole. A basic structure of formulas includes monarch, minister, assistant, and guide herbal medicines. According to TCM theory, so long as monarch herbal medicines and combination relationship of a classical prescription do not change, there is no change in the main clinical indications of the prescription [14]. In the procedure of TCM treatment, nearly all of the clinical prescriptions are modified by classic formulas [15]. In the prescription of CSS, Chinese Thorowax as monarch herbal medicine plays a principal role in therapeutic effect; Rhizoma Cyperi and Szechwan Lovage Rhizome are minister herbal medicine increasing the effect of Chinese Thorowax; Pericarpium Citri Reticulatae, Fructus Aurantii, and white peony root are used to harmonize the interaction between the ingredients; as a guide herbal medicine, licorice could guide the ingredients to the lesions. In the light of TCM theory, MSCP added Chinese Angelica or Radix Curcumae to the FD patients with qi stagnation and blood stasis, Cape Jasmine Fruit or Radix Scutellariae to the FD patients with transformation of depressed liver qi into fire, and Fructus Lycii or Radix Adenophorae to the FD patients with liver yin deficiency based on CSS [14]. Therefore, MCSP is now a popular traditional Chinese herbal formula for improving some symptoms similar to FD and recommended by functional dyspepsia traditional Chinese medicine diagnosis standard (2001 edition) [16].

The Study showed that MCSP could significantly increase propulsive rate of the small intestine (77.16 ± 3.42%) and decrease the residual amount of the pigment in the stomach in the rats [17]. Study from Qiu et al. suggested that ferulic acid and meranzin hydrate found in MCSP had the significant effect on promoting gastrointestinal motility in rats [18]. Saikosaponin (main activity of Chinese Thorowax) has anti-inflammatory activity and raised the painful threshold value [19]. Fructus Aurantii can significantly inhibit the spontaneous movement of isolated duodenum from rabbits and reduce the contraction force which presents concentration-response relationship [20]. Zhu et al.'s study proved that *Cyperus Rotundus* can delay gastric emptying, protecting gastric mucosa and reduce incidents of ulcer in the model of rats' gastric ulcer [21]. White Peony root can reduce internal high sensitivity and regulate the function of brain-gut axis [22, 23]. Animal studies have proved that Pericarpium Citri Reticulatae and licorice root promoted gastric emptying and small intestinal vermiculation and protected gastric mucosa [24].

### 1.2. Objectives

Evidence that clearly demonstrates effect and safety of MCSP has not yet been systematically studied. In this study, we evaluated the effects of MCSP in monotherapy or in combination with other prokinetic agents on FD through a rigorous systematic review and meta-analysis of randomized trial.

## 2. Methods

### 2.1. Eligibility Criteria

To make sure of the validity, applicability, and comprehensiveness, we specified the eligibility of inclusion and exclusion criteria for the review (Table 1). 

### 2.2. Information Sources

We searched the following electronic database: Cochrane Library (issue to August 2012), MEDLINE (1995 to August 2012), EMBASE (1995 to August 2012), SCI database (Science Citation Index Expanded), CNKI Database (China Knowledge Resource Integrated Database, 1979 to August 2012), Wanfang Data (1998 to August 2012), VIP Information (1985 to August 2012), CBMDisc (Chinese Biology Medical disc, August 2012), and Chinese Clinical Trials Registry (issue to August 2012). We also screened the relevant trials and identified review listed in the references. We restricted the language of publications to English and Chinese.

### 2.3. Search Strategy

We used the Boolean logic search for the databases as follows: (modified chaihu shugan *OR chaihu shugan *OR chai hu shu gan *OR Bupleurum Soothing*) and (functional dyspepsia OR nonulcer dyspepsia OR functional gastrointestinal disorders OR dyspepsia). 

### 2.4. Study Selection

Two reviewers (N. Yang and X. Qiu) independently screened the information contained in the title, abstract, key words, and description of each searched paper according to the inclusion and exclusion criteria. Any difference during assessment between the two reviewers was discussed or resolved by a third dependent reviewer (X. Jiang).

### 2.5. Data Collection Process

We developed a data extraction sheet for the included study. To avoid bias in the data abstraction, two reviewers (X. Qiu and Z. Hu) independently abstracted the data from the papers and compared the results. Disagreements were resolved by discussion between the two reviews; if no agreements could be reached, it was resolved by the third dependent reviewer (X. Jiang). 

### 2.6. Data Items

Items extracted from each study include citations of studies, method of the trials, simple size, gender and average age of the participants, treatment duration, each group's interventions, symptom improvement index and adverse drug reaction.

### 2.7. Risk of Bias

Two reviewers (N. Yang and Z. Hu) independently accessed the risk of bias for each trial according to the Cochrane Handbook for Systematic Reviewers of Interventions version 5.1.0 [48]. Cochrane collaboration addressed the following seven specific domains to describe the risk of bias, including random sequence generation, allocation concealment, blinding of participants and personnel, blinding of outcome assessment, incomplete outcome data, selective outcome reporting, and other biases. Each trail was categorized as “Low risk” of bias, “High risk” of bias, or “Unclear risk” of bias. Disagreements were resolved by discussion and by adjudicated by a third reviewer (Jiang) when necessary.

### 2.8. Summary Measures

Our comparisons included MCSP versus prokinetic drugs and MCSP plus prokinetic drugs versus prokinetic drugs. We analyzed the main outcomes data of the trials according to Cochrane Handbook. We reported risk ratio (RR) with 95% confidence intervals (CI) for the dichotomous data, and mean differences (MD) with 95% CI for continuous data. We used Chi-square statistic to assess the heterogeneity. Fixed effect model can be appropriate when there is statistical homogeneity (*P* > 0.1, *I* ^2^ < 50%) among the studies, and random effect model has to be pursued when statistical heterogeneity (*P* < 0.1, *I* ^2^ > 50%) exists in the trials. Publication bias was assessed by the funnel plot.

## 3. Results

### 3.1. Study Selection

**Figure 1:**
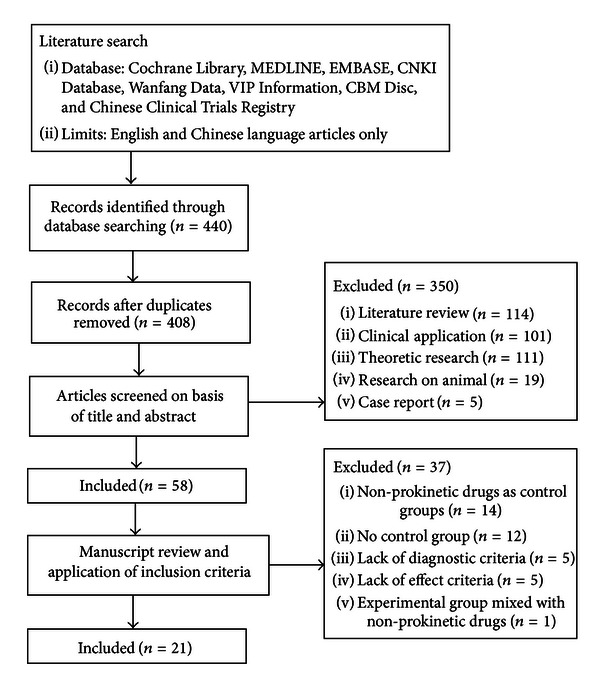
Flow diagram of selective for systematic review of MCSP for FD.

The study selection process, the reasons for excluding, and the search results at various stages were shown as a flow diagram (Figure 1) according to the planed search strategy. Successive rounds of review yielded 21 final studies, and one of those studies contained 2 RCTs [33]. The total of 22 RCTs were included, involving 1939 participants with FD. All studies were conducted in Chinese. In the trials, 13 RCTs compared MCSP versus prokinetic drugs, and 9 RCTs compared MCSP plus prokinetic drugs versus prokinetic drugs. There were no placebo controlled studies.

### 3.2. Study Characteristics

Of the 22 selected trials, 21 described the comparability analysis of source of participants, gender, age, and course of FD. The remaining 1 trail did not mention the information. The mean age of participants ranged from 33.9 to 56.0 years. Trial duration lasted for 3 weeks to 12 weeks. MCSP was prepared as decoction with traditional method of being boiled with water. All the interventions were taken orally. Further details of the included RCTs were presented in Table 1. Incidence of adverse reactions of 22 trials was no related reports. 

### 3.3. Risk of Bias within Studies

Overall the studies were at high risk of bias, which were shown in Table 2. All the trials claimed randomization, but only three RCTs [33, 38] reported that random number table was used. The remaining studies failed to provide information of how randomization was carried out. No allocation concealment and blinding were described. There were not notifications of dropouts and withdraws. No intention-to-treatment analyses were presented. 

### 3.4. Results of Individual Studies

#### 3.4.1. The Total Effective Rates of MCSP versus Prokinetic Drugs for FD

**Figure 2:**
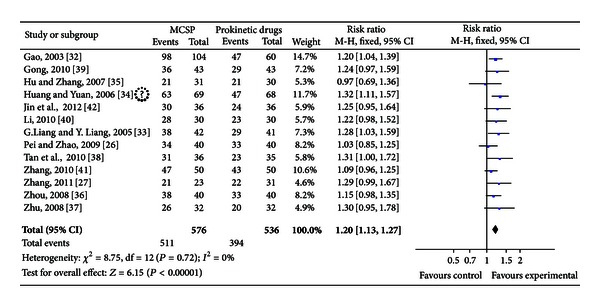
MCSP versus prokinetic drugs; outcomes: the total effectiveness.

Thirteen trials compared the clinical total effectiveness of MCSP versus prokinetic drugs for FD (*n* = 1112). The test for heterogeneity was insignificant statistically (*P* = 0.72, *I* ^2^ = 0%). Therefore, fixed effect model was used in the meta-analysis. The risk ratio for improvement of FD for MCSP treated versus prokinetic drugs treated was 1.20(95% CI 1.13 to 1.27), which achieved statistically significant (see Figure 2).

#### 3.4.2. The Total Effective Rates of MCSP Plus Prokinetic Drugs versus Prokinetic Drugs for FD

**Figure 3:**
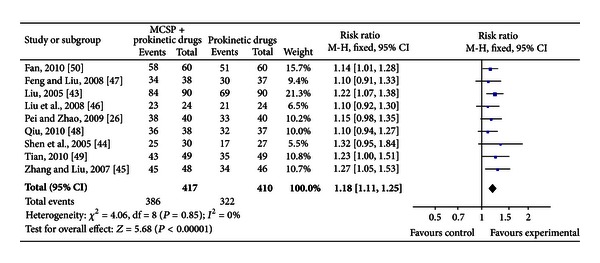
MCSP + prokinetic drugs versus prokinetic drugs; outcomes: the total effectiveness.

Nine studies compared the clinical total effectiveness of MCSP versus prokinetic drugs for FD (*n* = 827). The test for heterogeneity was insignificant statistically (*P* = 0.85, *I* ^2^ = 0%). Therefore, fixed effect model was used in the meta-analysis. MCSP plus prokinetic drugs had a greater probability of relieving the symptom of FD compared with prokinetic drugs alone (RR = 1.18, 95%, CI 1.11 to 1.25) (see Figure 3).

### 3.5. Risk of Bias across Studies

**Figure 4:**
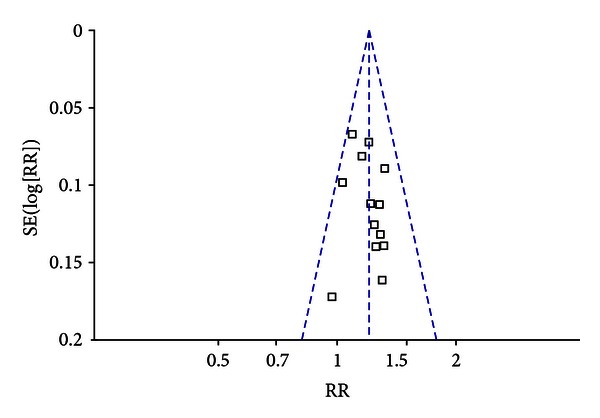
Funnel plot for MCSP versus prokinetic drugs for FD.

Figure 4 showed the reporting bias of trails on MCSP versus prokinetic drugs for FD. Each dot represented one study. The distribution of dots on the either side of center line was asymmetrical, which meant that there was a potential reporting bias.

To avoid distinguishing chance from real asymmetry because of fewer trials with too low power according to the Cochrane Handbook, we did not use test for funnel plot to detect the reporting biases of trails on MCSP plus prokinetic drugs versus prokinetic drugs for FD. 

## 4. Discussion

### 4.1. Summary of Evidence

With the development of new effective treatments, herbal medicines have been increasingly used in many countries especially for benign and chronic conditions such as FD [49, 50]. Some studies showed that artichoke leaf extract [51], peppermint and caraway oil [52], MCSP [27–47], and Rikkunshito (Liu Jun Zi Tang) [53] were advocated for FD. However, there had been no systematic research to indicate that MCSP did worse or better than other medicines against FD. 

FD is dyspepsia without evidence of an organic disease that is likely to explain the symptoms. There is no certain cure for it thus far. A vast number and variety of pharmacological treatment strategies was introduced to relieve the symptoms of FD. But some problems exist in allmost treatments. The efficacy of *H. pylori* eradication for FD remains controversial. Some meta-analyses concluded that H. pylori eradication had significant advantage over placebo [54, 55], but there were other studies which found insufficient or no benefit existing in treating FD [56, 57]. Histamine-type 2 receptor had superiority over placebo for patients with FD in clinical trials [13], however, which were merely limited to the symptom of epigastric pain and did not apply in global dyspepsia symptoms [58]. Some prokinetic agents showed more significant decrease in FD than placebo, which were widely prescribed in Canada, Mexico, and Australia like domperidone [13, 59]. But some of these such as metoclopramide and cisapride were of limited use because of the central nervous system and cardiac side-effects [60, 61]. Proton pump inhibitors (PPI) have been widely evaluated in the confined patients who have ulcer-like symptoms [62, 63]. Also antidepressants were reported to be used in treating FD, but there have been very limited data on it [64]. Thus treatment of patients with FD has been still a challenge and more effects should be made to develop new effective interventions. 

MCSP is based on an ancient formula that has been clinically used in China since 1600s. Since 1990s, published clinical trials have been reporting that MCSP has good therapeutic effects on FD. Our meta-analysis truly showed that MCSP might be a benefit for the patients suffering from FD. MCSP plus prokinetic drugs appeared to be more effective than prokinetic drugs alone. Although every ingredient of preparation does help to get rid of symptoms in FD, MCSP reflects the uncertainty about the clear mechanisms. It is believed that patients who are proved to be intractable to drug therapies likely suffer psychological disturbances [65]. A study supported that Chaihu Shugan powder was effective and safe in treating depression [66]. And the present meta-analysis proved significant effectiveness of MSCP in FD, which expressed a consistency between Western and Chinese medicine.

### 4.2. Limitations

There are several limitations in our study. Firstly, all the included trials were at high risk of bias. All the studies were in Chinese. Of the 23 trials, only two described the method of randomization, which weakened the reliability and repeatability of the research. None of the trails provided the information about allocation concealment and blinding. No multicenter and large-scale RCTs were identified. Sample size and allocation of samples among the groups are optional. Most of the literatures had no follow-up records. Lack of intention-to-treat analysis can also lead to biased judgment of efficacy. Secondly, except one study with treatment course of 3 weeks and another with 12 weeks, the length of course in the other included trials was 4 weeks. According to the diagnostic criteria for FD, it is a chronic condition with symptoms that recur frequently over time [16, 25, 26, 49]. Shortened therapeutic period of FD might impact the treatment and make it difficult to find adverse drug reactions. Moreover, included studies of MCSP did not change the monarch, minister, assistant, and guide herbal medicines of CSS's prescription, which only added several herbs, but it still needs experimental evidence to establish the effect of added ingredients. Last but not the least, MCSP in all of the included trials were prepared by boiling or decocting, which is traditional way of preparing herbal medicines in China. It contributed to no placebo used in clinical trials of traditional Chinese medicine. The composition of the same prescription, in fact, was flexible, and thus caused performance bias. Yet a research suggested that granules and decoction of 20 traditional Chinese formulas had no significant statistical difference in their effectiveness [67]. In the view of drug development, conventional forms of TCMs are beneficial for improving compliance and quality of clinical trials.

### 4.3. Conclusion

The result of this review provides preliminary data suggesting that either MCSP or MCSP plus prokinetic drugs achieved statistically significant improvement of symptoms of FD than prokinetic medications alone. However, the poor methodological quality made it difficult to determine the real role of MCSP in management of FD. After all, this review produced the rational evidence for the further use, research, and development of MCSP. Further large-scale high-quality clinical trials are required for assessment (see Table 3).

## Figures and Tables

**Table 1:** Inclusion and exclusion for the selected studies.

Inclusion criteria	Exclusion criteria
The patients diagnosed with FD according to Rome II [25], Rome III [26] consensus, or functional dyspepsia traditional Chinese medicine diagnosis standard [16]	Compared with other TCMs or control group combined with acid-suppressive drugs, eradication of *H. pylori*, fundus-relaxing drugs,and 5-HT_3 _receptor antagonists

Control group with prokinetic drugs	Successful treatment without measuring in terms of illness severity scores or the intensity of individual symptoms

Clearly outlined criteria for successful treatment	Course of treatment ≤ 2 weeks

Random allocation	

**Table 2:** Characteristics of randomized controlled trials of MCSP for functional dyspepsia.

Study	Method	Duration	*N* (M : F)	Mean age	Interventions	Symptom improvement
Gao, 2003 [27]	RCT, not blinded Comparison: individuals	4 w	164 (76 : 88)	37.3	(1) MCSP (bid) (2) Domperidone (10 mg, tid)	(1) TER: 94.2% (98/104) (2) TER: 78.3% (47/60)

G. Liang and Y. Liang, 2005 [28]	RCT, not blinded Comparison: individuals	4 w	83 (38 : 45)	39.6	(1) MCSP (bid) (2) Cisapride (10 mg, tid)	(1) TER: 90.5% (38/42) (2) TER: 70.7% (29/41)

Huang and Yuan, 2006 [29]	RCT, not blinded Comparison: individuals	4 w	137 (64 : 73)	40.4	(1) MCSP (bid) (2) Domperidone (10 mg, tid)	(1) TER: 92.5% (63/69) (2) TER: 70.6% (47/68)

Hu and Zhang, 2007 [30]	RCT, not blinded Comparison: individuals	12 w	61 (16 : 45)	39.5	(1) MCSP (bid) (2) Cisapride (5–10 mg, tid)	(1) TER: 67.7% (21/31) (2) TER: 70.0% (21/30)

Zhou, 2008 [31]	RCT, not blinded Comparison: individuals	4 w	80 (42 : 38)	52.4	(1) MCSP (bid) (2) Cisapride (5 mg, tid), Vitamin B6 (20 mg, tid), Oryzanol tablets (20 mg, tid)	(1) TER: 95.0% (38/40) (2) TER : 82.5% (33/40)

Zhu, 2008 [32]	RCT, not blinded Comparison: individuals	4 w	64 (28 : 36)	36.4	(1) MCSP (bid) (2) Domperidone (10 mg, tid)	(1) TER: 81.3% (26/32) (2) TER: 62.5% (20/32)

Pei and Zhao, 2009 [33]	RCT, not blinded Comparison: individuals	4 w	80 (41 : 39)	51.9	(1) MCSP (bid), (2) Trimebutine maleate tablets (100 mg, q.d)	(1) TER: 85.0% (34/40) (2) TER: 82.5% (33/40)

Tan et al., 2010 [34]	RCT, not blinded Comparison: individuals	4 w	71 (34 : 37)	36.5	(1) MCSP (bid) (2) Flupentixol melitracen tablets (2 pills, q.d)	(1) TER: 86.1% (31/36) (2) TER: 65.7% (23/35)

Gong, 2010 [35]	RCT, not blinded Comparison: individuals	4 w	86 (30 : 56)	36.8	(1) MCSP (bid) (2) Domperidone (10 mg, tid)	(1) TER: 83.7% (36/43) (2) TER: 65.1% (29/43)

Li, 2010 [36]	RCT, not blinded Comparison: individuals	4 w	60 (24 : 36)	40.6	(1) MCSP (bid) (2) Cisapride (5 mg, tid)	(1) TER: 93.3% (28/30) (2) TER: 76.7% (23/30)

Zhang, 2010 [37]	RCT, not blinded Comparison: individuals	3 w	100	N/A	(1) MCSP (bid) (2) Cisapride (5 mg, tid)	(1) TER: 94.0% (47/50) (2) TER: 86.0% (43/50)

Zhang, 2011 [38]	RCT, not blinded Comparison: individuals	4 w	54 (23 : 31)	N/A	(1) MCSP (bid) (2) Domperidone (10 mg, tid), Compound Azintamide tablets (2 pills, tid)	(1) TER: 91.3% (21/23) (2) TER: 70.9% (22/31)

Jin et al., 2012 [39]	RCT, not blinded Comparison: individuals	4 w	72 (32 : 40)	39.3	(1) MCSP (bid) (2) Mosapride citrate tablets (5 mg, tid)	(1) TER: 83.3% (30/36) (2) TER: 66.7% (24/36)

Liu et al., 2005 [40]	RCT, not blinded Comparison: individuals	4 w	180 (72 : 108)	47.5	(1) MCSP (bid), Cisapride (5 mg, tid) (2) Cisapride (5 mg, tid)	(1) TER: 93.3% (84/90) (2) TER: 76.7% (69/90)

Shen et al., 2005 [41]	RCT, not blinded Comparison: individuals	4 w	57 (26 : 31)	33.9	(1) MCSP (bid), Cisapride (10 mg, tid) (2) Cisapride (10 mg, tid)	(1) TER: 83.3% (25/30) (2) TER: 62.9% (17/27)

Zhang and Liu, 2007 [42]	RCT, not blinded Comparison: individuals	4 w	94 (38 : 56)	56.0	(1) MCSP (bid), Mosapride (5 mg, tid) (2) Mosapride (5 mg, tid)	(1) TER: 93.8% (45/48) (2) TER: 73.9% (34/46)

Liu et al., 2008 [43]	RCT, not blinded Comparisonindividuals	4 w	48 (19 : 29)	35.0	(1) MCSP (bid), Flupentixol melitracen tablets (1 pill, q.d) (2) Flupentixol melitracen tablets (1 pill, q.d)	(1) TER: 95.8% (23/24) (2) TER: 87.5% (21/24)

Feng and Liu 2008 [44]	RCT, not blinded Comparison: individuals	4 w	75 (33 : 42)	36.1	(1) MCSP (bid), Domperidone (10 mg, tid) (2) Domperidone (10 mg, tid)	(1) TER: 89.5% (34/38) (2) TER: 81.1% (30/37)

Pei and Zhao, 2009 [33]	RCT, not blinded Comparison: individuals	4 w	80 (42 : 38)	52.3	(1) MCSP (bid), Trimebutine maleate tablets (100 mg, q.d) (2) Trimebutine maleate tablets (100 mg, q.d)	(1) TER: 95.0% (38/40) (2) TER: 82.5% (33/40)

Qiu, 2010 [45]	RCT, not blinded Comparison: individuals	4 w	75 (28 : 47)	43.5	(1) MCSP (bid), Domperidone (10 mg, tid) (2) Domperidone (10 mg, tid)	(1) TER: 94.7% (36/38) (2) TER : 86.5% (32/37)

Tian, 2010 [46]	RCT, not blinded Comparison: individuals	4 w	98 (43 : 55)	40.9	(1) MCSP (bid), Domperidone (10 mg, tid) (2) Domperidone (10 mg, tid)	(1) TER: 87.8% (43/49) (2) TER : 71.4% (35/49)

Fan, 2010 [47]	RCT, not blinded Comparison: individuals	1 m	60 (21 : 39)	46.5	(1) MCSP (bid), Cisapride (5 mg, tid) (2) Cisapride (5 mg, tid)	(1) TER : 96.7% (58/60) (2) TER : 85.0% (51/60)

RCT: randomized clinical trial; F: female; m: male; w: week; M: month; 1: experimental group; 2: control group; TER: total effective rate.

**Table 3:** Assessment of risk of bias of included studies.

Study	Random sequence generation	Allocation concealment	Blinding of participants and personnel	Blinding of outcome assessment	Incomplete outcome data	Selective reporting	Other biases
Gao, 2003 [27]	U	H	U	U	U	U	H
G. Liang and Y. Liang, 2005 [28]	U	H	U	U	U	U	H
Huang and Yuan, 2006 [29]	U	H	U	U	U	U	H
Hu and Zhang, 2007 [30]	U	H	U	U	U	U	H
Zhou, 2008 [31]	U	H	U	U	U	U	H
Zhu, 2008 [32]	U	H	U	U	U	U	H
Pei and Zhao, 2009 [33]	L	H	U	U	U	U	H
Tan et al., 2010 [34]	U	H	U	U	U	U	H
Gong, 2010 [35]	U	H	U	U	U	U	H
Li, 2010 [36]	U	H	U	U	U	U	H
Zhang, 2010 [37]	L	H	U	U	U	U	H
Zhang, 2011 [38]	U	H	U	U	U	U	H
Jin et al., 2012 [39]	U	H	U	U	U	U	H
Liu, 2005 [40]	U	H	U	U	U	U	H
Shen et al., 2005 [41]	U	H	U	U	U	U	H
Zhang and Liu, 2007 [42]	U	H	U	U	U	U	H
Liu et al., 2008 [43]	U	H	U	U	U	U	H
Feng and Liu 2008 [44]	U	H	U	U	U	U	H
Pei and Zhao, 2009 [33]	L	H	U	U	U	U	H
Qiu, 2010 [45]	U	H	U	U	U	U	H
Tian, 2010 [46]	U	H	U	U	U	U	H
Fan, 2010 [47]	U	H	U	U	U	U	H

L: low risk of bias; U: unclear; H: high risk of bias.
